# Rotational Particle Separation in Solutions: Micropolar Fluid Theory Approach

**DOI:** 10.3390/polym13071072

**Published:** 2021-03-29

**Authors:** Vladimir Shelukhin

**Affiliations:** 1 Lavrentyev Institute of Hydrodynamics, 630090 Novosibirsk, Russia; shelukhin@list.ru; 2 Novosibirsk State University, 630090 Novosibirsk, Russia

**Keywords:** suspensions, micro-polar fluids, yield stress

## Abstract

We develop a new mathematical model for rotational sedimentation of particles for steady flows of a viscoplastic granular fluid in a concentric-cylinder Couette geometry when rotation of the Couette cell inner cylinder is prescribed. We treat the suspension as a micro-polar fluid. The model is validated by comparison with known data of measurement. Within the proposed theory, we prove that sedimentation occurs due to particles’ rotation and rotational diffusion.

## 1. Introduction

The classical water-based drilling muds contain only water and clay and their performances are generally poor. Polymers used in drilling fluids improve stability and cutting removal. Currently, the polymer-based drilling fluids represent 15 to 18% of the total cost (about 1 million) of petroleum well drilling [1]. The reason is that such fluids appear to have load carrying capabilities, or, in other words, a yield stress, associated with the solid-like state and which primarily arises from the colloidal forces between the smallest suspended particles. Furthermore, in such systems, when the agitation is increased, a fluid-like state is recovered. Even in the fluid state, these materials usually show highly nonlinear behavior. The complex rheology related to the change of the internal state in the suspensions is still rather poorly understood.

Due to scale separation between colloidal and non-colloidal particles, polymer-based drilling muds with cuttings and other materials (concrete casting, foodstuff transport, etc.) can be considered as suspensions of noncolloidal particles embedded in a yield stress fluid. Substantial progress in the understanding of the behavior of such materials can thus be made by studying the impact of adding noncolloidal particles to a yield stress fluid of known properties [2]. Here, we develop a new mathematical model for suspensions embedded in a polymer-based fluid. To validate the approach, we consider rotation flows between two concentric cylinders when the external cylinder is fixed and the inner one rotates with a prescribed speed. The principal feature of such flows is the particles’ migration toward the external boundary.

It is proved by Svedberg [3] that the particles’ migration effect occurs also in pure colloidal suspensions. Moreover, sedimentation happens at high rotations. Currently, Svedberg’s ultracentrifugation is known as an effective tool for studies of interaction between macromolecules of colloidal systems [4]. In this method, a highly disperse colloidal solution is enclosed in a wedge-shape cell rotating about an axis coinciding with the apex of the wedge [5]. Samples are centrifuged at speeds to produce sedimentation and shallow concentration gradients. A great progress in the study of such a flow was achieved by applying the diffusive Lamm equation based on the special empirical sedimentation coefficient [6]. Here, we restrict ourself to rotation flows of suspensions between two concentric cylinders paying attention to comparison with known laboratory experiments. We use methods of mechanics of a continuum by applying conservation laws only and not involving the concept of sedimentation coefficient inherent in the Lamm equation. However, we also obtain the sedimentation effect.

We take into account particles rotation and rotational diffusion. To this end, we apply the theory of micropolar fluids which allows for particles rotation and microinertia. According to this theory, which is a part of rational mechanics, any infinitesimal volume contains sufficiently many particles. This is why such an approach is good for colloidal solutions. However, the micropolar fluid theory turned out to be useful in the study of suspension of noncolloidal particles and also including granular fluids. As is proved in [7], it is due to particle rotation that the Segré–Silberberg effect occurs [8]. Such an effect is known as a tubular pinch phenomenon stating that particles tend to migrate towards a concentric annular region for the laminar flow of neutrally buoyant dilute suspension of rigid spheres through a circular tube.

Important sectors handling granular fluids include civil engineering (bitumen, concrete, embankments, ballast trains, soil stability), mining (extraction, transport), the chemical industry (fuel and catalysts are often deployed in the form of grains in order to maximize the surface of exchange), the pharmaceutical industry (from the handling of powders for the manufacture of medicine to the handling of drugs themselves) and the food industry (animal food, cereals), to name but a few.

Results from laboratory experiments, numerical simulations, and theoretical approaches from other fields have enriched and renewed our understanding of granular fluids. In many rheological papers on shear flows, particle rotation is ignored. Instead, the authors apply the theory which states that microstructural non-homogeneous particles distribution is due to anisotropy. To this end, the rheological equations involve not only shear stress but normal stress differences as well [9]. The micro-polar fluid theory stands out among other approaches since it handles particle rotations and micro-inertia effects within the mechanics of continua. Such a theory finds many applications in granular flows [10], electrorheology [11,12], ferrofluids [13], visco-elastic micro-polar fluids [14], and liquid crystals [15]. Some results concern blood rheology [16,17,18]. Particularly, explanations were provided for phenomena including the Fåhræus–Lindqvist effect, the Fåhræus effect, and the plasma skimming effect [19,20,21,22]. Any new theory involves additional unknown rheological constants. This restricts applications and further progress. In the micro-polar theory, one such constant is a relative viscosity. What is it? First, we comment on the ordinary fluid viscosity.

In simple shear flows of typical Newtonian or non-Newtonian fluids, viscosity $\eta_{s}\left[Pa\cdot s\right]$ is given by the formula $\eta_{s}=$ $\tau /\dot{\gamma }$ or

(1) $$\tau =\eta_{s}\dot{\gamma },$$

where τ is the shear stress and $\dot{\gamma }$ is the shear rate. The rates’ definition will be given below.

In the micro-polar fluid theory allowing for internal spins, stress tensor loses symmetry, couple stress appears, and the angular momentum equation should be included into conservation laws. Instead of Equation (1), one writes for simple shear flows the following equation:

(2) $$\tau =\sqrt{{\left(\eta_{s}\dot{\gamma }\right)}^{2}+{\left(\eta_{sk}{\dot{\gamma }}_{a}\right)}^{2}},$$

where ${\dot{\gamma }}_{a}$ is a shear rate related to particle rotation, and $\eta_{sk}$ is an additional viscosity. In what follows, we call $\eta_{sk}$ skew-symmetrical viscosity motivated by rheological relationships which will be discussed later.

When the volume concentration of particles ϕ is equal to zero, the viscosity $\eta_{s}$ of the interstitial fluid is assumed to be known. Clearly, $\eta_{s}$ is the usual shear viscosity and it is well known how it can be measured. The skew-symmetric viscosity manifests itself when ϕ>0, and poor knowledge of it hampers applications of the micro-polar fluid models. The above description of possible applications supports the view that it is of importance to know how skew-symmetric viscosity depends on the particle concentration. The goal of the first sections of the present paper is to get a better insight of how $\eta_{sk}$ depends on ϕ. In Section 1, Section 2, Section 3, Section 4 and Section 5, we validate some formula for the skew-symmetric viscosity and apply it in Section 6 to the problem of particles migration for the steady flows of yield stress granular fluids in a concentric-cylinder Couette geometry when rotation of the Couette cell inner cylinder is prescribed.

## 2. Micro-Polar Fluids

Here, we remind notions concerning a micropolar fluid within the theory of the Cosserat continuum. Such a fluid exhibits micro-rotational effects and micro-rotational inertia; the fluid can support couple stresses and body couples and possesses a non-symmetric stress tensor. The theory of micro-polar fluids goes back to [23,24,25], where gyration tensor, inertial spin, conservation of micro-inertia, and objectivity of micro-deformation rate tensors are derived and discussed. For an overview of developed theories, we refer the reader to [26,27].

In the Cosserat continuum [28], each material point is treated as a rigid body in the following sense. To such a point with the Lagrange coordinates (t,ξ), one can assign a position vector x(t,ξ) in the three-dimensional Euclidean space and three orthogonal directors $d_{i}\left(t,\xi \right)$, i=1,2,3. Rotation of the vectors $d_{i}$ is governed by a rotation orthogonal tensor Q(t,ξ). The rotation velocity tensor

$$\Omega \left(t,x\right)=Q_{t}Q^{*}$$

is skew-symmetric, and it enjoys the representation formula

$$\Omega \cdot h=\omega \times h\;\forall \;h\in R^{3},\;{\left(\Omega \cdot h\right)}_{i}\equiv \Omega_{ij}h_{j},$$

where ω(t,x) is the micro-rotational velocity vector,

$$2\omega =e_{i}\times \left(\Omega \cdot e_{i}\right)=\epsilon :\Omega .$$

Here, ${\left\{e_{i}\right\}}_{1}^{3}$ is any orthogonal basis in $R^{3}$, and ϵ is the Levi–Civita third order tensor,

$$\epsilon \langle a,b,c\rangle =a\cdot \left(b\times c\right),\;e_{i}\times e_{j}=\epsilon_{sij}e_{s},\;\epsilon_{sij}\equiv \epsilon \langle e_{s},e_{i},e_{j}\rangle ,\;{\left(\epsilon :\Omega \right)}_{i}\equiv \epsilon_{ijk}\Omega_{jk}.$$

Given a 3×3-matrixes *A* and *B*, we use the notation $A^{*}$ for the adjoint matrix such that

$$a\cdot \left(A\cdot b\right)=b\cdot \left(A^{*}\cdot a\right)\;\forall a,b\in R^{3},\;{\left(A^{*}\right)}_{ij}=A_{ji},$$

and the scalar product A:B is defined by A:B= $A_{ij}B_{ij}$.

With v(t,x) standing for the velocity of the mass center of the Cosserat material point (t,ξ), the micropolar fluid is characterized by two rates of strain tensors *B* and *A*:

(3) B=∇v−Ω,A=∇ω.

Here, we use the notations ${\left(\nabla v\right)}_{ij}=$ $\partial v_{i}/\partial x_{j}$, ${\left(\nabla v\right)}_{ij}^{*}=$ $\partial v_{j}/\partial x_{i}$. An instant stress state of such a fluid is characterized by the couple stress tensor N(t,x) in addition to the Cauchy stress tensor T(t,x). Let *S* stand for the viscous part of the stress tensor, T=−pI+S. In what follows, we use the symmetric and skew-symmetric parts of a matrix *D*:

$$D_{s}=\frac{D+D^{*}}{2},\;D_{a}=\frac{D-D^{*}}{2}.$$

In the typical stress–strain relation

(4) $$T=-pI+2\eta_{s}\cdot {\left(\nabla v\right)}_{s},$$

of Newtonian or non-Newtonian fluids, the scalar factor $\eta_{s}$ is the viscosity, *p* is the pressure, with *I* and *T* being the identity and stress tensors. The tensor *T* is symmetric as is well known in the classical fluid mechanics theory, i.e., $T^{*}=T$. Such a symmetry results from momentum and angular momentum laws. On the other hand, the angular momentum law is valid automatically if we postulate symmetry of *T*. This is why nobody invokes such a law in applications.

We remind readers that the constitutive laws of a simple micropolar fluid are [26]

(5) $$T=-pI+S,\;S=2\eta_{s}B_{s}+2\eta_{sk}B_{a},\;N=2\gamma A,$$

where $\eta_{s}$, $\eta_{sk}$ are the viscosities and γ is angular viscosity. The first rheological equation in (5) suggests that the contributions of the symmetric part $B_{s}={\left(\nabla v\right)}_{s}$ and skew-symmetric part $B_{a}$ of the rate of strain tensor *B* into local stress state are different. It is proved in [25] that both the rate of strain tensors *B* and *A* are objective.

Let us introduce the relative angular velocity

$$\omega_{r}=\omega -rot\;v/2.$$

Observe that in fluid mechanics shear stress and shear rates in (1) are defined as follows:

$$\tau =\sqrt{S:S/2},\;\dot{\gamma }=\sqrt{2B_{s}:B_{s}},\;{\dot{\gamma }}_{a}=\sqrt{2B_{a}:B_{a}}=2\left|\omega_{r}\right|.$$

Due to the identity $2{\left(\nabla v\right)}_{a}\cdot h=$ rotv×h ∀h, we have

$$B_{a}\cdot h=-\omega_{r}\times h\;\forall h.$$

Thus, the skew-symmetric viscosity characterizes how relative micro-rotations contribute into the local stress state.

Observe that in the Cosserat continua the Cauchy stress tensor is not symmetric and the vector

$$t=e_{i}\times \left(T\cdot e_{i}\right)=\epsilon :T$$

is a stress symmetry defect measure in the sense that the equality t=0 implies $T^{*}=T$ and vice versa. By definition of t, we have the formula

(6) t·ω=T:Ω.

The momentum and the angular momentum laws are

(7) $$\frac{\partial (\rho v)}{\partial t}+\mathrm{div}\left(\rho v⊗v\right)=-\nabla p+\mathrm{div}\;S+\rho f,\;$$

(8) $$J\left(\frac{\partial (\rho \omega )}{\partial t}+\mathrm{div}\left(\omega ⊗\rho v\right)\right)=\mathrm{div}\;N-\epsilon :S,$$

where ρ is the density, *J* is the micro-inertia scalar, f is the mass force vector and

$${\left(\mathrm{div}\;N\right)}_{i}\equiv \partial N_{ij}/\partial x_{j},\;{\left(\omega ⊗v\right)}_{ij}=\omega_{i}v_{j}.$$

The density ρ satisfies the mass conservation law

(9) $$\dot{\rho }+\rho \mathrm{div}\;v=0.$$

## 3. Skew-Symmetrical Viscosity of Dilute Suspensions of Rigid Particles

Let us consider Couette-like steady flows of suspensions between two parallel planes in the *x*-direction when the upper plane y=h is fixed and the lower plane y=0 moves in the *x*-direction with the velocity *V*. The volume particle concentration ϕ is assumed to be fixed.

We outline the method for determination of the skew-symmetrical viscosity $\eta_{sk}$. Assume that the stress applied to the moving plate can be measured. By continuity, one can tell the fluid stress in the nearby fluid region. On the other hand, one can calculate such a fluid stress by one or another mathematical model. First, we determine the stress $S_{1}^{m}$ at the moving plane by applying the micro-polar fluid theory. Then, we calculate the stress $S_{1}^{v}$ at the moving plane by applying the Navier–Stokes theory. We equate the two stresses and derive the skew-symmetrical viscosity $\eta_{sk}$ from the equality $S_{1}^{m}=$ $S_{1}^{v}$.

Let us first treat the suspension as a micropolar fluid with the prescribed volume particle concentration ϕ. The above assumptions upon the flows suggest that the velocity vector v, the micro-rotational velocity vector ω and the pressure *p* depend on the vertical variable *y* only:

$$v=v\left(y\right){\left(1,0,0\right)}^{T},\;\omega =\omega \left(y\right){\left(0,0,1\right)}^{T},\;\;p=p\left(y\right),\;0<y<h.$$

For such flows, the matrices ∇v and Ω become

$$\nabla v=\left(\begin{array}{ccc} 0 & v_{y} & 0\\ 0 & 0 & 0\\ 0 & 0 & 0 \end{array}\right),\;\Omega =\left(\begin{array}{ccc} 0 & -\omega & 0\\ \omega & 0 & 0\\ 0 & 0 & 0 \end{array}\right),\;\frac{\partial v}{\partial y}=v_{y}.$$

Hence,

$$B_{s}=\left(\begin{array}{ccc} 0 & v_{y}/2 & 0\\ v_{y}/2 & 0 & 0\\ 0 & 0 & 0 \end{array}\right),\;B_{a}=\left(\begin{array}{ccc} 0 & v_{y}/2+\omega & 0\\ -v_{y}/2-\omega & 0 & 0\\ 0 & 0 & 0 \end{array}\right)$$

Projections of the momentum and the angular momentum equations on the *x* and *z*-directions become

(10) $$0=\frac{\partial S_{12}}{\partial y},$$

(11) $$0=\frac{\partial N_{32}}{\partial y}+S_{21}-S_{12},$$

respectively, where the tensor components are given by the formulas

$$S_{21}=e_{y}\cdot S\langle e_{x}\rangle ,\;S_{12}=e_{x}\cdot S\langle e_{y}\rangle ,\;N_{32}=e_{z}\cdot N\langle e_{y}\rangle .$$

Constitutive laws (5) take the form

(12) $$S_{12}=\left(\eta_{s}+\eta_{sk}\right)\frac{\partial v}{\partial y}+2\eta_{sk}\omega ,\;S_{21}=\left(\eta_{s}-\eta_{sk}\right)\frac{\partial v}{\partial y}-2\eta_{sk}\omega .$$

We note that system (10) and (11) does not contain pressure. As is well known, it can be restored from the momentum equation projected on the *y*-direction.

The viscosity $\eta_{sk}\left(\phi \right)$ vanishes when ϕ→0. The same is true for the relative viscosity

$$\varepsilon \left(\phi \right)=\frac{\eta_{sk}\left(\phi \right)}{\eta_{s}},$$

which we represent via the expansion series

$$\varepsilon \left(\phi \right)=\Lambda \phi +\Lambda_{2}\phi^{2}+\cdots .$$

Whereas the velocity v satisfies the no-slip boundary conditions, we require that

(13) ω=α(ϕ)rotv/2aty=0andy=h,

where $\alpha \left(\phi \right)=\alpha_{0}\phi$. The latter condition implies that the micro-rotations agree with macro-rotations at the boundary [29]. For the Couette-like flows, we arrive at the following boundary-value problem in the domain 0<y<h:

(14) $$\frac{\partial }{\partial y}\left[\left(1+\varepsilon \left(\phi \right)\right)\frac{\partial v}{\partial y}+2\varepsilon \left(\phi \right)\omega \right]=0,$$

(15) $$2\gamma \frac{\partial^{2}\omega }{\partial y^{2}}-2\eta_{s}\varepsilon \left(\phi \right)\left(\frac{\partial v}{\partial y}+2\omega \right)=0,$$

(16) $${v|}_{y=0}=V,\;v{{|}_{y=h}=0,\;\omega |}_{y=0,h}=-\frac{\alpha_{0}\phi }{2}\frac{\partial v}{\partial y}{|}_{y=0,h}.$$

We solve the boundary value problem (14)–(16) looking for (v,w) as the asymptotic expansion series

(17) $$v\left(y,\phi \right)=v^{0}\left(y\right)+v^{1}\left(y\right)\phi +\cdots ,\;\omega \left(y,\phi \right)=\omega^{0}\left(y\right)+\omega^{1}\left(y\right)\phi +\cdots .$$

Setting these series in (14)–(16), one can write each equality in (14)–(16) in the form

$$\phi^{0}{\left(\cdots \right)}_{0}+\phi^{1}{\left(\cdots \right)}_{1}+\cdots =0.$$

The coefficients $v^{i}$ and $\omega^{j}$ are determined from the conditions ${\left(\cdots \right)}_{k}=0$ for any *k*.

Particularly, if k=0, we derive the following boundary value problems for the functions $v^{0}\left(y\right),\omega^{0}\left(y\right)$:

(18) $$\frac{\partial^{2}v^{0}}{\partial y^{2}}=0,\;v^{0}{{|}_{y=0}=V,\;v^{0}|}_{y=h}=0,$$

(19) $$0=\frac{\partial^{2}\omega^{0}}{\partial y^{2}},\;\omega^{0}{{|}_{y=0}=\omega^{0}|}_{y=h}=0.$$

Similarly, we find that the function $v^{1}$ satisfies the boundary value problem

(20) $$\frac{\partial }{\partial y}\left[\frac{\partial v^{1}}{\partial y}+\Lambda \left(\frac{\partial v^{0}}{\partial y}+2\omega^{0}\right)\right]=0,\;v^{1}{{|}_{y=0}=v^{1}|}_{y=h}=0.$$

Solving these problems, we find that

$$\omega^{0}=0,\;v^{1}=0,\;v^{0}=V\left(1-y/h\right).$$

Starting from the definitions (12) related to the micro-polar fluid theory, we can write the expansion series for the stress $S_{12}\left(\phi \right)$ as follows:

$$S_{12}\left(\phi \right)=S_{12}^{0}+S_{12}^{1}\phi +o\left(\phi \right).$$

Clearly,

$$S_{12}^{0}=\eta_{s}\frac{\partial v^{0}}{\partial y},\;S_{12}^{1}=\eta_{s}\frac{\partial v^{1}}{\partial y}+\eta_{s}\Lambda \left(\frac{\partial v^{0}}{\partial y}+2\omega^{0}\right).$$

Now, we can calculate the relative stress at the moving plane:

(21) $$\frac{S_{12}^{m}\left(\phi \right)}{S_{12}^{m}\left(0\right)}\equiv \frac{S_{12}\left(\phi \right)}{S_{12}\left(0\right)}{|}_{y=0}=1+\phi \Lambda +o\left(\phi \right).$$

It is assumed that both the stresses $S_{12}^{m}\left(\phi \right)$ and $S^{m}{\left(0\right)}_{12}$ are measured at the same velocity *V* of the moving plate.

Let us consider flows within the same Couette geometry, starting from the Navier–Stokes theory. In such a theory, the stress tensor *S* is symmetric. Denoting $S=S_{12}$, one can find the velocity v(y) by solving the boundary-value problem

(22) $$0=\frac{\partial S}{\partial y},\;S=\eta \left(\phi \right)\frac{\partial v}{\partial y},\;v{{|}_{y=0}=V,\;v|}_{y=h}=0,$$

where η(ϕ) is the effective viscosity.

Let $S_{1}^{v}$ stand for the stress *S* at the moving plate, $S_{1}^{v}=$ ${S|}_{y=0}$. Given $S_{1}^{v}$, one can derive from (22) the following formula for the apparent viscosity η(ϕ):

(23) $$\eta \left(\phi \right)=-\frac{hS_{1}^{v}}{V}$$

Observe that $S_{1}^{v}$ has negative values since v(y) is a decreasing function of *y*. It follows from (23) that

(24) $$\frac{\eta (\phi )}{\eta (0)}=\frac{S_{1}^{v}\left(\phi \right)}{S_{1}^{v}\left(0\right)},\;\eta \left(0\right)\equiv \eta_{s}.$$

It is assumed that both the stresses $S_{1}^{v}\left(\phi \right)$ and $S_{1}{\left(0\right)}^{v}$ are measured at the same velocity *V* of the moving plate.

For dilute suspensions, the left-hand side of (24) is given by the Einstein formula [30]

(25) $$\frac{\eta (\phi )}{\eta_{s}}=1+E\phi +o\left(\phi \right),\;E≃2.5.$$

We equate the relative stresses: $S_{12}^{m}\left(\phi \right)/S_{12}^{m}\left(0\right)=$ $S_{1}^{v}\left(\phi \right)/S_{1}^{v}\left(0\right)$. Now, it follows from (21), (24) and (25) that

1+Eϕ=1+ϕΛ+o(ϕ).

Hence, Λ=E. By the above arguments, we conclude that the skew-symmetric viscosity for dilute suspensions satisfies the representation formula

(26) $$\eta_{sk}\left(\phi \right)/\eta_{s}=E\phi +o\left(\phi \right),$$

where E≃2.5 is the Einstein factor.

**Remark** **1.** *By the same asymptotic arguments, we can conclude that for the Couette flows between two concentric cylinders formula (26) becomes*

(27) $$\eta_{sk}\left(\phi \right)/\eta_{s}=G\cdot E\phi +o\left(\phi \right),$$

*where G is the geometrical factor equal to ${\left(R_{2}/R_{1}\right)}^{2}$, with $R_{1}$ being the smaller radius.*

One more conclusion from the above arguments is that there is a correlation between the Navier–Stokes theory and the micro-polar fluid theory:

(28) $$\eta \left(\phi \right)/\eta \left(0\right)=1+\eta_{sk}\left(\phi \right)/\eta_{s}+o\left(\phi \right),\;\eta_{s}=\eta \left(0\right),$$

where $\eta_{s},\eta_{sk}$ are the micro-polar fluid viscosities of the suspension and η(ϕ) is the apparent viscosity of the same suspension described by the Navier–Stokes rheology. The law (28) is verified by the asymptotic series argument for dilute suspensions, with the left-hand side given by the Einstein law η(ϕ)= η(0)(1+Eϕ)+o(ϕ).

On the other hand, there is an extended Krieger–Douhgerty empirical closure [31]

(29) $$\eta \left(\phi \right)/\eta \left(0\right)={\left(1-\phi /\phi^{*}\right)}^{-E\phi^{*}},\;E=2.5,$$

for dense suspensions, where $\phi^{*}$ is a maximal volume concentration. Such a closure suggests that, by setting (29) in (28), we can define the skew-symmetric viscosity $\eta_{sk}\left(\phi \right)$ as follows:

(30) $${\left(1-\phi /\phi^{*}\right)}^{-E\phi^{*}}=1+\eta_{sk}\left(\phi \right)/\eta_{s}.$$

In the next sections, we verify this empirical formula by studying flows of dense suspensions paying attention to particle rotation.

## 4. Flows of Suspensions of Rigid Particles in the Herschel–Bulkley Fluid

By the theoretical approach of Chateau et al. [32], Ovarlez et al. [9] showed that the flows of yield stress suspensions in a concentric-cylinder Couette geometry can be modeled by a Herschel–Bulkley behavior of same index as their interstitial fluid. The theory was proved to be in an agreement with the laboratory experiments based on the magnetic resonance imaging techniques [9].

We are going to address the same experiments as in [9] to verify formula (30). First, we extend the constitutive laws (5) to allow for the yield stress rheology. According to [33], the Cosserat–Bingham fluid rheology is defined as follows:

(31) $$\begin{array}{ccc} S & = & \left\{\begin{array}{cc} 2\eta_{s}B_{s}+2\eta_{sk}B_{a}+\tau_{*}\frac{B_{0}}{|B_{0}|}, & if\;B(x,t)\neq 0,\\ S_{p}\left(x,t\right), & if\;B(x,t)=0, \end{array}\right. \end{array}$$

(32) $$\begin{array}{ccc} N & = & \left\{\begin{array}{cc} 2\gamma A+\tau_{n}\frac{A}{\left|A\right|}, & if\;A(x,t)\neq 0,\\ N_{p}\left(x,t\right), & if\;A(x,t)=0, \end{array}\right. \end{array}$$

where

$$B_{0}=B_{s}+\varepsilon B_{a},\;\varepsilon \left(\phi \right)=\frac{\eta_{sk}\left(\phi \right)}{\eta_{s}}$$

and $\tau_{*}$ and $\tau_{n}$ are yield stresses; the unknown plug tensors $S_{p}$ and $N_{p}$ obey the restrictions

$$|S_{p}|\leq \tau_{*},\;\left|N_{p}\right|\leq \tau_{n}.$$

It is proved in [34] that the formulation (31) is equivalent to the inclusion $S\in \partial V_{*}\left(B_{0}\right)$, where the scalar potential $V_{*}\left(D\right)$ is defined for any matrix $D\in R^{3\times 3}$ by the formula $V_{*}\left(D\right)=\eta_{s}{\left|D\right|}^{2}+\tau_{*}\left|D\right|$. We remind that the subdifferential formulation $S\in \partial V_{*}\left(B_{0}\right)$ implies that

$$S:\left(D-B_{0}\right)\leq V_{*}\left(D\right)-V_{*}\left(B_{0}\right)\;for\;all\;D\in R^{3\times 3}.$$

Similarly, the constitutive law (32) is equivalent to the inclusion $N\in \partial V_{n}\left(A\right)$ with $V_{n}\left(D\right)={\gamma |D|}^{2}+\tau_{n}\left|D\right|$, $\forall D\in R^{3\times 3}$. The meaning of the plug zone $\left|N\left(x,t\right)\right|\leq \tau_{n}$ is discussed in [35].

Let $T_{0}$ be a characteristic time. We denote the dimensionless second invariant of the rate of strain tensor $B_{0}$ by *I*: $I=T_{0}\left|B_{s}\right|$. To transform the Cosserat–Bingham fluid constitutive laws (31) and (32) into the Cosserat–Herschel–Bulkley fluid rheological equations, we assume that

(33) $$\eta_{s}=\eta_{s0}I^{n-1}\;and\;\frac{\eta_{sk}\left(\phi \right)}{\eta_{s}}=\varepsilon \left(\phi \right),\;where\;\varepsilon \left(\phi \right)={\left(1-\phi /\phi^{*}\right)}^{-E\phi^{*}}-1.$$

Observe that the classical Hershel–Bulkley model results from the constitutive laws (31)–(33) if the particle volume concentration ϕ vanishes.

We consider steady axially symmetric flows of a suspension between two coaxial cylinders centered on the *z*-axis. The inner cylinder of the radius $R_{1}$ rotates with the angular velocity $\Omega_{0}\left[s^{-1}\right]$ and the external cylinder of the radius $R_{2}$ is fixed. The volume particle concentration ϕ is assumed invariable along the radial coordinate. The case of variable ϕ will be addressed in Section 6.

In what follows, we use the unit vectors $e_{r}$, $e_{\varphi }$, $e_{z}$ of the cylindrical coordinate system. The assumption of axially symmetry of flows suggests that the velocity vector v, the micro-rotational velocity vector ω and the pressure *p* depend on the radial variable *r* only:

(34) $$v=v\left(r\right)e_{\varphi },\;\omega =\omega \left(r\right)e_{z},\;p=p\left(r\right),\;\mathrm{rot}\;v=\left(\frac{\partial v}{\partial r}+\frac{v}{r}\right)e_{z},\;\mathrm{rot}\;\omega =-\frac{\partial \omega }{\partial r}e_{\varphi }.$$

For such flows, the matrices ∇v and Ω in the cylindrical coordinate system become

$$\nabla v=\left(\begin{array}{ccc} 0 & -v/r & 0\\ \partial v & 0 & 0\\ 0 & 0 & 0 \end{array}\right),\;\Omega =\left(\begin{array}{ccc} 0 & -\omega & 0\\ \omega & 0 & 0\\ 0 & 0 & 0 \end{array}\right),\;\nabla \omega =\left(\begin{array}{ccc} 0 & 0 & 0\\ 0 & 0 & 0\\ \partial \omega & 0 & 0 \end{array}\right),$$

where we denoted ∂v/∂r by ∂v for simplicity. Here,

$${\left(\nabla v\right)}_{ij}=e_{i}\cdot v^{\prime }\left(x\right)\langle e_{j}\rangle ,\;i=\left(r,\varphi ,z\right),\;v^{\prime }\left(x\right)\langle a\rangle =\frac{d}{d\lambda }v\left(x+\lambda a\right){|}_{\lambda =0}.$$

Hence,

$$B=\left(\begin{array}{ccc} 0 & -v/r+\omega & 0\\ \partial v-\omega & 0 & 0\\ 0 & 0 & 0 \end{array}\right),\;$$

$$B_{s}=\left(\begin{array}{ccc} 0 & \frac{\partial v-v/r}{2} & 0\\ \frac{\partial v-v/r}{2} & 0 & 0\\ 0 & 0 & 0 \end{array}\right),\;B_{a}=\left(\begin{array}{ccc} 0 & \omega -\frac{\partial v+v/r}{2} & 0\\ \frac{\partial v+v/r}{2}-\omega & 0 & 0\\ 0 & 0 & 0 \end{array}\right)$$

As for the rate of strain tensor *A*, we have that $A_{zr}=\partial \omega /\partial r$ and $A_{ij}=0$, otherwise. Projections of the momentum Equation (7) and the angular momentum law (8) on the vectors $e_{\varphi }$ and $e_{z}$ become

(35) $$0=\frac{\partial S_{\varphi r}}{\partial r}+\frac{S_{\varphi r}+S_{r\varphi }}{r},$$

(36) $$0=\frac{\partial N_{zr}}{\partial r}+\frac{N_{zr}}{r}+S_{\varphi r}-S_{r\varphi },$$

respectively, where the tensor components are given by the formulas

$$S_{\varphi r}=e_{\varphi }\cdot S\langle e_{r}\rangle ,\;S_{r\varphi }=e_{r}\cdot S\langle e_{\varphi }\rangle ,\;N_{zr}=e_{z}\cdot N\langle e_{r}\rangle .$$

Given a vector e, we apply the notation ${\left(S\langle e\rangle \right)}_{i}=$ $S_{ij}e_{j}$. Observe that the other components of *S* and *N* are equal to zero. In what follows, we use the equation

(37) $$\frac{\rho v^{2}}{r}=\frac{\partial p}{\partial r},$$

resulting from projection of the momentum equation (7) onto the vector $e_{r}$.

We calculate that

$$I=2^{-1/2}T_{0}\sqrt{{\left(\partial v/\partial r-v/r\right)}^{2}+\varepsilon^{2}{\left(\partial v/\partial r+v/r-2\omega \right)}^{2}}.$$

The constitutive laws (31) and (31) become

(38) $$S_{r\varphi }=\left(2\eta_{s}+\frac{\tau_{*}T_{0}}{I}\right)\left[\frac{1}{2}\left(\frac{\partial v}{\partial r}-\frac{v}{r}\right)-\frac{\varepsilon }{2}\left(\frac{\partial v}{\partial r}+\frac{v}{r}-2\omega \right)\right]\;if\;I\neq 0,$$

(39) $$S_{\varphi r}=\left(2\eta_{s}+\frac{\tau_{*}T_{0}}{I}\right)\left[\frac{1}{2}\left(\frac{\partial v}{\partial r}-\frac{v}{r}\right)+\frac{\varepsilon }{2}\left(\frac{\partial v}{\partial r}+\frac{v}{r}-2\omega \right)\right]\;if\;I\neq 0,$$

(40) $$|S_{r\varphi }{|}^{2}+{\left|S_{\varphi r}\right|}^{2}\leq \tau_{*}^{2}\;if\;I=0,$$

(41) $$N_{zr}=2\gamma \frac{\partial \omega }{\partial r}+\tau_{n}sign\;\left(\frac{\partial \omega }{\partial r}\right)\;if\;\frac{\partial \omega }{\partial r}\neq 0,\;\left|N_{zr}\right|\leq \tau_{n}\;if\;\frac{\partial \omega }{\partial r}=0,$$

The boundary condition (13) for the angular velocity ω and the no-slip condition for *v* become

(42) $${\omega |}_{r=R_{i}}=\frac{\alpha_{0}\phi }{2}\left(\frac{\partial v}{\partial r}+\frac{v}{r}\right){|}_{R_{i}},\;v{{|}_{r=R_{1}}=R_{1}\Omega ,\;v|}_{r=R_{2}}=0.$$

To study numerically the boundary-value problem (33)–(42) in the annulus $R_{1}<r<R_{2}$, we pass to dimensionless variables:

$$r^{\prime }=\frac{r}{R_{1}},\;v^{\prime }=\frac{v}{V},\;\omega^{\prime }=\frac{\omega }{\omega_{0}},\;S_{r\varphi }^{\prime }=\frac{S_{r\varphi }}{S^{0}},\;S_{\varphi r}^{\prime }=\frac{S_{\varphi r}}{S^{0}},\;N_{zr}^{\prime }=\frac{N_{zr}}{N_{0}},\;\gamma_{1}=\frac{\gamma }{R_{1}^{2}\eta_{s0}},$$

with

$$V=R_{1}\Omega_{0},\;\omega_{0}=\Omega_{0},\;T_{0}=\frac{1}{\Omega_{0}}\left[s\right],\;S_{0}=\eta_{s0}\Omega_{0},\;\tau_{*1}=\frac{\tau_{*}}{\eta_{s0}\Omega_{0}},\;\tau_{n1}=\frac{\tau_{n}R_{1}}{\gamma \Omega_{0}},\;N_{0}=R_{1}\eta_{s0}\Omega_{0}.$$

Observe that the dimensionless yield stress $\tau_{*1}$ is the inverse of the Bingham number for the Couette flows:

$$\tau_{*1}=\frac{1}{Bn},\;Bn=\frac{\eta_{s0}\Omega_{0}}{\tau_{*}}.$$

In new variables,

(43) $$I=2^{-1/2}\sqrt{{\left(\partial^{\prime }v^{\prime }/\partial r^{\prime }-v^{\prime }/r^{\prime }\right)}^{2}+\varepsilon^{2}{\left(\partial^{\prime }v^{\prime }/\partial r^{\prime }+v^{\prime }/r^{\prime }-2\omega^{\prime }\right)}^{2}},\;\frac{\eta_{s}}{\eta_{s0}}=I^{n-1}.$$

(44) $$S_{r\varphi }^{\prime }=\left[\frac{1}{2}\left(\frac{\partial^{\prime }v^{\prime }}{\partial r^{\prime }}-\frac{v^{\prime }}{r^{\prime }}\right)-\frac{\varepsilon }{2}\left(\frac{\partial^{\prime }v}{\partial r^{\prime }}+\frac{v^{\prime }}{r^{\prime }}-2\omega^{\prime }\right)\right]\left(2I^{n-1}+\frac{\tau_{*1}}{I}\right)\;if\;I\neq 0,$$

(45) $$S_{\varphi r}^{\prime }=\left[\frac{1}{2}\left(\frac{\partial^{\prime }v^{\prime }}{\partial r^{\prime }}-\frac{v^{\prime }}{r^{\prime }}\right)+\frac{\varepsilon }{2}\left(\frac{\partial^{\prime }v^{\prime }}{\partial r^{\prime }}+\frac{v^{\prime }}{r^{\prime }}-2\omega^{\prime }\right)\right]\left(2I^{n-1}+\frac{\tau_{*1}}{I}\right)\;if\;I\neq 0,$$

(46) $$|S_{r\varphi }^{\prime }{|}^{2}+{\left|S_{\varphi r}^{\prime }\right|}^{2}\leq \tau_{*1}^{2}\;if\;I=0,$$

(47) $$N_{zr}^{\prime }=\gamma_{1}\left(2\frac{\partial^{\prime }\omega^{\prime }}{\partial r^{\prime }}+\tau_{n1}sign\;\frac{\partial^{\prime }\omega^{\prime }}{\partial r^{\prime }}\right)\;if\;\frac{\partial^{\prime }\omega^{\prime }}{\partial r^{\prime }}\neq 0,\;\left|N_{zr}^{\prime }\right|\leq \tau_{n1}\;if\;\frac{\partial^{\prime }\omega^{\prime }}{\partial r^{\prime }}=0,$$

(48) $$0=\frac{\partial^{\prime }S_{\varphi r^{\prime }}^{\prime }}{\partial r^{\prime }}+\frac{S_{\varphi r}^{\prime }+S_{r\varphi }^{\prime }}{r^{\prime }},$$

(49) $$0=\frac{\partial^{\prime }N_{zr}^{\prime }}{\partial r^{\prime }}+\frac{N_{zr}^{\prime }}{r^{\prime }}+S_{\varphi r}^{\prime }-S_{r\varphi }^{\prime },$$

(50) $$\omega^{\prime }{|}_{r^{\prime }=1,a}=\frac{\alpha_{0}\phi }{2}\left(\frac{\partial^{\prime }v^{\prime }}{\partial r^{\prime }}+\frac{v^{\prime }}{r^{\prime }}\right){|}_{r^{\prime }=1,a},\;v^{\prime }{{|}_{r^{\prime }=1}=1,\;v^{\prime }|}_{r^{\prime }=a}=0.$$

## 5. Skew-Symmetric Viscosity versus Particles Concentration

Here, we apply the mathematical model developed in the previous section to justify formula (30) for the skew-symmetric viscosity. To perform calculations, we fix parameters of the interstitial Hershel–Bilkley fluid. Rheological constitutive law of such a fluid results from (31) by setting ϕ=0:

(51) $$\begin{array}{ccc} S & = & \left\{\begin{array}{cc} 2\eta_{s}B_{s}+\tau_{*}\frac{B_{s}}{|B_{s}|}, & if\;B_{s}\left(x,t\right)\neq 0,\\ S_{p}\left(x,t\right), & if\;B_{s}\left(x,t\right)=0, \end{array}\right.\;where\;\eta_{s}=\eta_{s0}{\left(T_{0}|B_{s}|\right)}^{n-1}. \end{array}$$

We denote

(52) $$\tau_{y}=\sqrt{2}\tau_{*},\;\eta_{HB}=2^{5/4}\eta_{s0}T_{0}^{-1/2}$$

and set n=1/2, $R_{1}=4\left[\mathrm{cm}\right]$, $R_{2}=6\left[\mathrm{cm}\right]$. Then, it follows from (51) that

(53) $$\sqrt{2S:S}=\tau_{y}+\eta_{HB}{\left(\sqrt{2B_{s}:B_{s}}\right)}^{1/2}.$$

The concentrated emulsion obeying Equation (53) was considered in [9] with $\tau_{y}=$ 22 [Pa] and $\eta_{HB}=$5.3 [Pa$\cdot s^{1/2}$]. Given the angular velocity Ω [rpm] of the rotating inner cylinder, we calculate that $T_{0}=1/\Omega_{0}=$ (60/Ω)[s]. We consider the same fluid as in [9], hence one can define the consistency $\eta_{s0}$ and the yield stress $\tau_{*}$ as follows:

(54) $$\eta_{s0}=2^{-5/4}\eta_{HB}\left[\mathrm{Pa}\cdot s^{1/2}\right]{\left(\frac{60}{\Omega }\right)}^{1/2}\left[s^{1/2}\right],\;\tau_{*}=\tau_{y}/\sqrt{2}\left[\mathrm{Pa}\right].$$

Observe that consistency depends on Ω because of the special choice the characteristic time $T_{0}$ and the definition of the dimensionless invariant *I* of the rate of strain tensor $B_{s}$.

It is proved in [35] that the rotation yield stress $\tau_{n}$ causes the appearance of clusters of particles, with each cluster being a plug zone which rotates as a rigid body. Conglomerates of particles were not observed in [9] for the Couette flows of suspensions between two rotating cylinders; this is why $\tau_{n}$ and $\tau_{n1}$ can be neglected. As for the dimensionless angular viscosity $\gamma_{1}$ and the boundary-value dimensionless parameter $\alpha_{0}$, we variate them to fit experimental data.

Approximate solutions of the system (43)–(50) can be obtained by regularization [36]. Given a small positive δ, we substitute the dimensionless invariant *I* in (44) and (45) by $I_{\delta }$, where

$$I_{\delta }=2^{-1/2}\sqrt{{\left(\partial^{\prime }v^{\prime }/\partial r^{\prime }-v^{\prime }/r^{\prime }\right)}^{2}+\varepsilon^{2}{\left(\partial^{\prime }v^{\prime }/\partial r^{\prime }+v^{\prime }/r^{\prime }-2\omega^{\prime }\right)}^{2}+\delta^{2}},$$

with δ↘0.

First, we tune the model (43)–(50) by setting ϕ=0 and addressing the pure interstitial Herschel–Bulkley fluid with $\tau_{y}\left(0\right)=$ 22[Pa] and $\eta_{HB}\left(0\right)=$ $5.3[\mathrm{Pa}\cdot s^{1/2}]$ as in [9]. Equations become

(55) $$I=2^{-1/2}\sqrt{{\left(\partial^{\prime }v^{\prime }/\partial r^{\prime }-v^{\prime }/r^{\prime }\right)}^{2}},\;\frac{\eta_{s}}{\eta_{s0}}=I^{n-1},\;n=1/2.$$

(56) $$S_{r\varphi }^{\prime }=S_{\varphi r}^{\prime }=\frac{1}{2}\left(\frac{\partial^{\prime }v^{\prime }}{\partial r^{\prime }}-\frac{v^{\prime }}{r^{\prime }}\right)\left(2I^{n-1}+\frac{\tau_{*1}}{I}\right)\;if\;I\neq 0,$$

(57) $$2|S_{r\varphi }^{\prime }{|}^{2}\leq \tau_{*1}^{2}\;if\;I=0,$$

(58) $$0=\frac{\partial^{\prime }S_{\varphi r^{\prime }}^{\prime }}{\partial r^{\prime }}+\frac{2S_{\varphi r}^{\prime }}{r^{\prime }},$$

(59) $$v^{\prime }{{|}_{r^{\prime }=1}=1,\;v^{\prime }|}_{r^{\prime }=R_{2}/R_{1}}=0.$$

Observe that in such a case the model (55)–(59) depends on one parameter $\tau_{*1}\left(0\right)$ only. Given the angular velocity $\Omega [s^{-1}]$, we find from (52) the value of the Bingham number Bn(0) by the formula

(60) $$Bn\left(0\right)=\frac{\eta_{HB}\left(0\right)\Omega_{0}^{1/2}}{2^{3/4}\tau_{y}\left(0\right)}.$$

**Figure 1:**
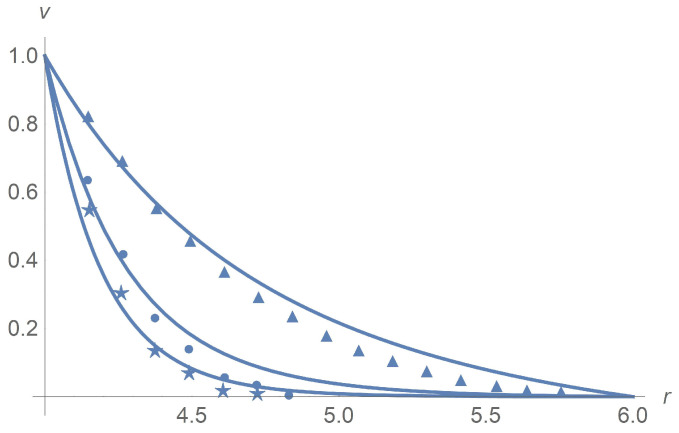
Calculated dimensionless velocity profiles (solid lines) versus the radial variable for pure interstitial Herschel–Bulkley fluid without particles, ϕ=0. The lines from the bottom upward correspond to Ω=2, Ω=5 and Ω=100, respectively. Stars, balls, and triangles stand for measurement data [9] in the cases Ω=2, Ω=5 and Ω=100, respectively.

**Figure 2:**
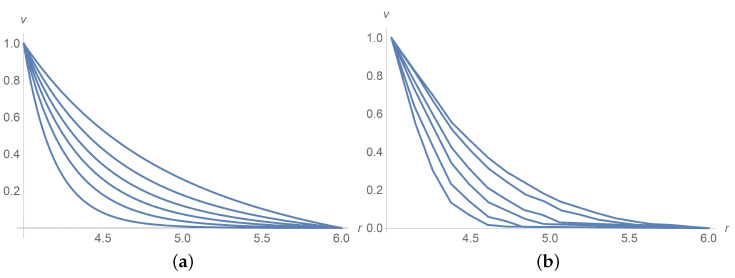
Dimensionless velocity profiles versus the radial variable in pure interstitial Herschel–Bulkley fluid without particles, ϕ=0, for Ω=2,5,10,20,50,100[rpm] from the bottom upwards. (**a**) calculations, (**b**) measured data [9].

Figure 1 and Figure 2 depict very good agreement of calculation results for ϕ=0 with experiment data [9] for different angular velocities $\Omega_{0}\left[s^{-1}\right]$ but in the case of passage to the effective Bingham number $Bn_{e}\left(0\right)$:

(61) $$Bn_{e}\left(0\right)=1.5\cdot Bn\left(0\right),$$

We think that such a discrepancy between the measured and effective Bingham numbers is due to the following reasons. Real 3D-flows are described by 1D-equations. The gravitation, the height of the annulus region, and the lateral boundaries effect are not taken into account. It may be that viscoelastic fluid property is also of importance, which calls for more adequate modeling.

Figure 1 corresponds to Ω=2,Ω=5 and Ω=100[rpm]. The same agreement between calculation results and experiment data is observed for Ω=10,20 and 50[rpm], but we omit pictures to save the space. Figure 2a combines all velocity profiles for Ω=2,5,10,20,50 and 100[rpm]; it fits the laboratory experiments exposed in Figure 2b. Why does the velocity profile become less steep as Ω increases? Equations (60) and (61) answer the question. In fact, there is a motionless plug zone of the Herschel–Bulkley fluid near the exterior cylinder. Our approximate solutions based on the regularization approach do not catch the plug zone well. The bigger the plug zone, the steeper the velocity curve. However, the dimensionless yield stress $\tau_{*1}\left(0\right)$ stipulates the size of the plug zone and, at the same time, it varies inversely with the angular velocity Ω.

Now, we consider suspensions assuming that they can be modeled by a Herschel–Bulkley behavior of the same index as their interstitial fluid, with their consistency and their yield stress depending on the particle volume fraction. To determine the function $\tau_{*1}\left(\phi \right)$ in Equations (55)–(59), we apply correlations proposed in [9] for n=1/2:

(62) $$\frac{\eta_{HB}\left(\phi \right)}{\eta_{HB}\left(0\right)}={\left(\frac{\tau_{y}\left(\phi \right)}{\tau_{y}\left(0\right)}\right)}^{3/2}\cdot {\left(1-\phi \right)}^{-1/2},$$

(63) $$\frac{\tau_{y}\left(\phi \right)}{\tau_{y}\left(0\right)}=\sqrt{\left(1-\phi \right){\left(1-\phi /\phi^{*}\right)}^{-2.5\phi^{*}}}$$

In our notations (see (33)), equality (63) becomes

$$\frac{\tau_{y}\left(\phi \right)}{\tau_{y}\left(0\right)}=\sqrt{\left(1-\phi )(\varepsilon (\phi )-1\right)}.$$

It follows from the definitions (60) and (61) that

(64) $$\frac{\tau_{*1}\left(\phi \right)}{\tau_{*1}\left(0\right)}={\left(\frac{1-\phi }{1+\varepsilon (\phi )}\right)}^{1/4}.$$

**Figure 3:**
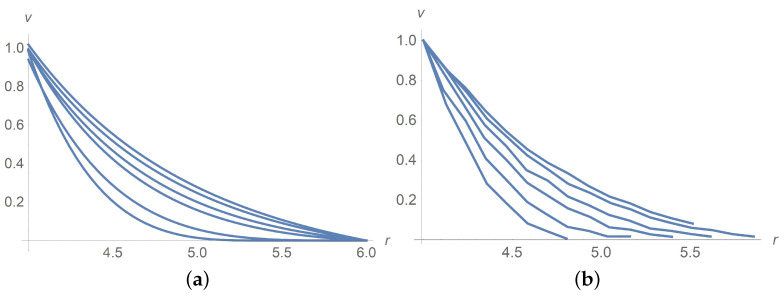
Dimensionless velocity profiles of the Herschel–Bulkley fluid with the apparent $\eta_{HB}\left(\phi \right)$ and $\tau_{y}\left(\phi \right)$ for ϕ=0.3 and Ω=2,5,10,20,50,100[rpm] from the bottom upwards. (**a**) calculations, (**b**) measured data [9].

With the function $\tau_{*1}\left(\phi \right)$ given by (64), we solve Equations (55)–(59) and find an agreement with experiment data [9]. Calculations and laboratory data depicted in Figure 3 are related to ϕ=0.3 for Ω= 2,5,10,20,50 and 100[rpm].

Let us return to the general system (43)–(50) which describes micropolar fluid. We fix $\tau_{*1}$ by Equations (64), (60) and (61). To choose the dimensionless parameters $\gamma_{1}$ and $\alpha_{0}$, we apply the method of least squares based on minimizing the function

$$F\left(\gamma_{1},\alpha_{0}\right)=\sum_{i,j,k}{\left|v_{\gamma_{1},\alpha_{0}}^{\prime }\left(r_{i},\phi_{j},\Omega_{k}\right)-v_{data}^{\prime }\left(r_{i},\phi_{j},\Omega_{k}\right)\right|}^{2}.$$

Here, $v_{data}^{\prime }$ is the measured velocity at different locations $r_{i}$ for different volume concentrations $\phi_{j}$ and different angular velocities $\Omega_{k}$, $v_{\gamma_{1},\alpha_{0}}^{\prime }\left(r,\phi ,\Omega \right)$ is the calculated velocity, with $\gamma_{1}$ and $\alpha_{0}$ being prescribed. Calculations reveal that the optimal $\gamma_{1}$ and $\alpha_{0}$ take values $\gamma_{1}^{*}=10.93$ and $\alpha_{0}^{*}=0.79$ provided $\tau_{y}\left(0\right)=$ 22[Pa] and $\eta_{HB}\left(0\right)=$ $5.3[Pa\cdot s^{1/2}]$. Below, we provide results of calculations with the chosen data $\tau_{*1}$, $\gamma_{1}^{*}$ and $\alpha_{0}^{*}$.

**Figure 4:**
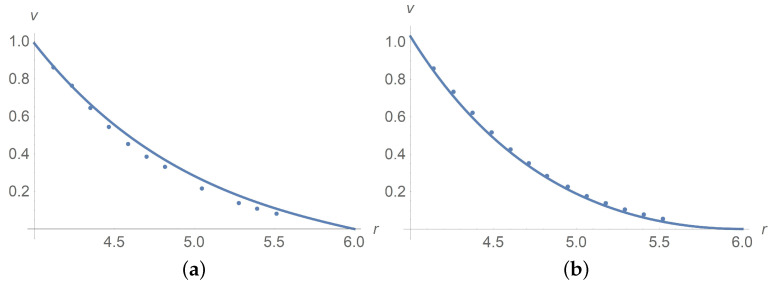
The solid line corresponds to a dimensionless velocity profile versus the radial variable for Ω=100[rpm]. Dots stand for experimental data [9]. (**a**) ϕ=0.3, (**b**) ϕ=0.1.

**Figure 5:**
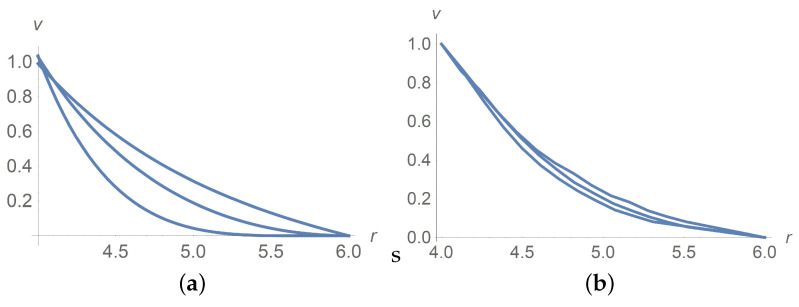
Dimensionless velocity profiles for Ω=100[rpm]. The curves from the bottom upwards correspond to ϕ=0, ϕ=0.1 and ϕ=0.3. (**a**) Calculations, (**b**) measured data [9].

**Figure 6:**
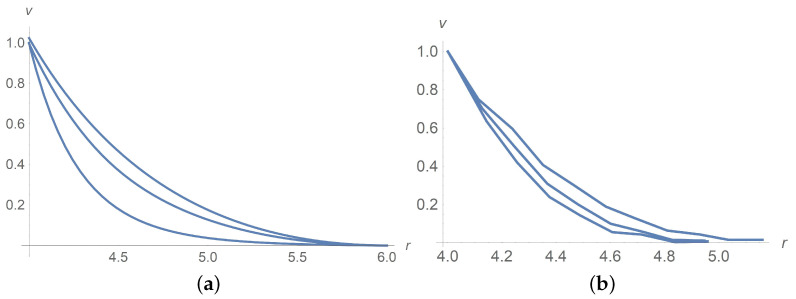
Dimensionless velocity profiles for Ω=5[rpm]. The curves from the bottom upwards correspond to ϕ=0,0.1 and 0.3. (**a**) calculations, (**b**) measured data [9].

**Figure 7:**
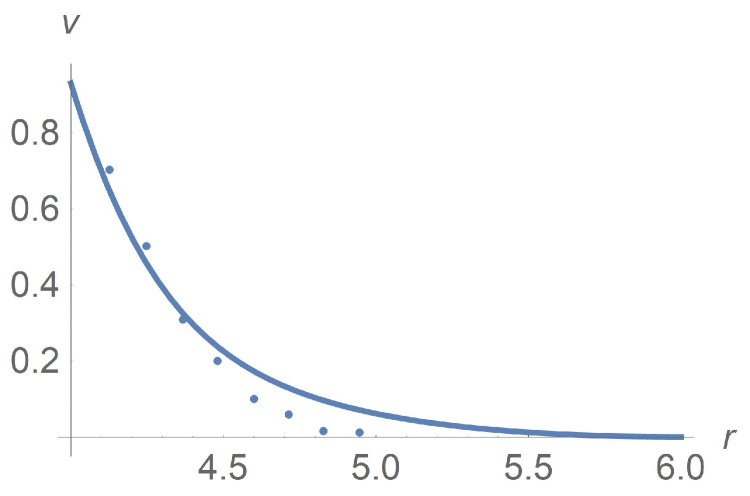
The solid line corresponds to dimensionless velocity profile versus the radial variable for Ω=5[rpm] and ϕ=0.1. Dots stand for experimental data [9].

**Figure 8:**
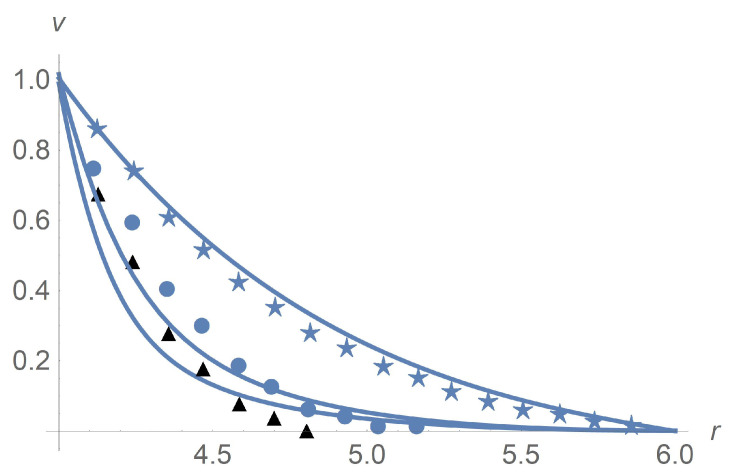
Calculated dimensionless velocity profiles versus the radial variable for ϕ=0.3. The lines from bottom upward correspond to Ω=2[rpm], Ω=5[rpm] and Ω=50[rpm], respectively. Triangles, balls and stars are the measured data from [9] corresponding to Ω=2[rpm], Ω=5[rpm] and Ω=50[rpm], respectively.

**Figure 9:**
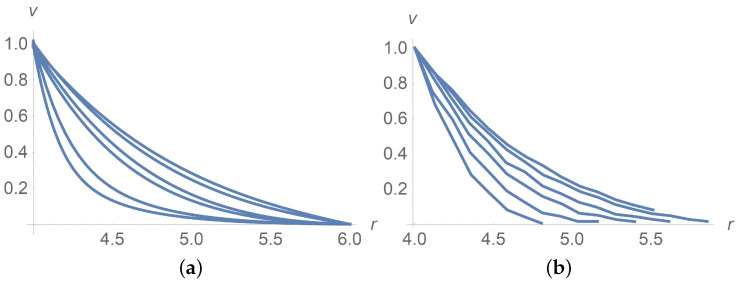
(**a**) Calculated dimensionless velocity profiles for ϕ=0.3. The curves correspond to Ω=2,5,10,20,50,100[rpm] from the bottom upwards. (**b**) Measured [9] dimensionless velocity profiles of the Herschel–Bulkley fluid with ϕ=0.3 and Ω taking values 2,5,10,20,50,100[rpm] from the bottom upwards.

Figure 4 and Figure 5a concern calculations for Ω=100[rpm] when ϕ takes values 0,0.1 and 0.3. Measured data in Figure 5b borrowed from [9] confirm agreement with calculations. The same is true for Ω=5[rpm] when ϕ takes values 0,0.1 and 0.3 as shown in Figure 6 and Figure 7. In Figure 8 and Figure 9, ϕ is fixed equal to 0.3 with Ω taking on the values 2,5,10,20,50, and 100[rpm]. Calculations agree with the measured data from [9].

**Figure 10:**
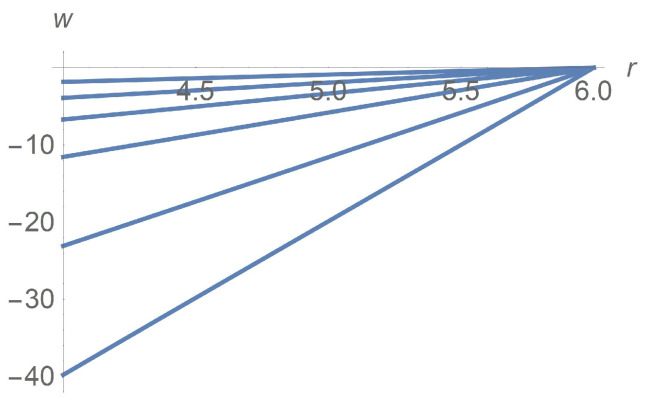
Calculated angular velocity w[rpm] profiles for ϕ=0.3. The curves correspond to Ω=2,5,10,20,50,100[rpm] from the top down.

The micro-polar fluid rheology equations (5) predict particle rotation. Figure 10 depicts profiles of the dimension angular velocity w(r) when ϕ is fixed equal to 0.3 with Ω taking on the values 2,5,10,20,50, and 100[rpm].

Although the dimensionless angular viscosity $\gamma_{1}=$ $2^{5/4}\gamma R_{1}^{-2}\eta_{HB}^{-1}\left(\phi \right)\Omega$ is determined, we can not identify the dimensional angular viscosity γ. Indeed, the tuning step (61) implies that we substituted $\tau_{y}\left(0\right)/\eta_{HB}\left(0\right)$ by $0.2\cdot \tau_{y}\left(0\right)/\eta_{HB}\left(0\right)$. However, in doing so, it is impossible to know individual reduced values both of $\tau_{y}\left(0\right)$ and $\eta_{HB}\left(0\right)$. Hence, we don’t know the reduced value of $\eta_{HB}\left(\phi \right)$.

Let us comment on some discrepancy between calculations and data of measurement. One can see in Figure 4b and Figure 7 that, with increasing the rotational velocity at a constant volume concentration ϕ=0.1, the theoretical results are shown to better agree with experiment. It is a problem of calculations. The reason is that we consider the viscoplastic fluid and the plug zone (with zero velocity and low shear stress) near the external cylinder being larger at low rotations. The governing system of equations becomes degenerate in such a zone. Mathematical theory of degenerate systems of differential equations and corresponding numerical methods built into Wolfram Mathematica are not well developed yet. There is one more difficulty related to the Hershel–Bulkley fluid viscosity (with the power n=1/2) becoming infinite when the velocity gradient vanishes somewhere. To get over these difficulties, we apply the regularization technique and substitute the invariant *I* of the rate of strain tensor $B_{0}$ in Equations (38)–(40) by its non-vanishing approximation $I_{\delta }$.

A comparison of the results in Figure 1 (upper curve), and Figure 4 at the rotational velocity Ω=100 rpm shows that, with increasing the volume concentration, the calculated results better agree with experiments at intermediate concentrations ϕ=0.1; at lower (ϕ = 0) and at higher concentrations (ϕ = 0.3), the deviations increase. The reason is that there are no data of measurement for the dimensionless parameters $\gamma_{1}$ and $\alpha_{0}$. To choose them, we apply the method of least squares based on minimizing the functional $F(\gamma_{1},\alpha_{0})$. As it happened, a discrepancy between the measured data and calculations for different concentrations and angular velocities is due to the optimal choice of these unknown parameters.

The above calculations confirm that Equation (30) for the skew-symmetric viscosity $\eta_{sk}\left(\phi \right)$ can be of use.

Observe that comparison with experiments for colloidal suspensions is contained in Figure 1, Figure 2 and Figure 5 since, in the case ϕ=0, the suspension becomes a pure colloidal fluid.

## 6. Rotational Sedimentation

Here, we consider more general mathematical model allowing for non-uniform particle distribution. We introduce the mass concentration of particles as follows:

(65) $$c=\frac{{\bar{\rho }}_{s}\phi }{\rho },\;\rho ={\bar{\rho }}_{s}\phi +{\bar{\rho }}_{f}\left(1-\phi \right),$$

where ρ is the total density, ${\bar{\rho }}_{s}$ is the particle density and ${\bar{\rho }}_{f}$ is the density of the interstitial fluid. Given *c*, one can restore from (65) the volume concentration and the total density by the formulas

(66) $$\phi =\frac{{\bar{\rho }}_{f}c}{{\bar{\rho }}_{f}c+{\bar{\rho }}_{s}\left(1-c\right)},\;\rho \left(c\right)=\frac{{\bar{\rho }}_{f}{\bar{\rho }}_{s}}{{\bar{\rho }}_{f}c+{\bar{\rho }}_{s}\left(1-c\right)}.$$

Due to these formulas, any given function of the volume concentration like the relative viscosity ε(ϕ) can be defined in terms of the mass concentration *c*. It is explained in [7] that *c* satisfies the conservation law

(67) $$\frac{\partial (\rho c)}{\partial t}+\mathrm{div}\left(\rho cv+l\right)=0,$$

where l is the concentration flux obeying the generalized Fick equation

(68) $$\rho l=-D\nabla c-D_{p}\nabla p+D_{\omega }\mathrm{rot}\;\omega \times \omega_{r}.$$

The scalar parameters *D*$[cm^{2}/s],$$D_{p}$$\left[cm^{3}\cdot s/g\right]$, and $D_{\omega }$$[cm^{2}\cdot s],$ stand for the diffusion, barodiffusion, and spin diffusion coefficients.

Observe that, instead of (5), the couple stress tensor *N* is prescribed by the rheological equation

(69) $$N=2\gamma A+\frac{D_{\omega }}{2}\epsilon :\left(l\times \omega_{r}\right)$$

to meet the entropy production law [7] where the skew-symmetric matrix ϵ:a is defined by the formula

$${\left(\epsilon :a\right)}_{ij}=a_{k}\epsilon_{ikj},\;a=a_{i}e_{i},$$

in any orthogonal basis ${\left\{e_{i}\right\}}_{1}^{3}$. It is due to spin diffusion that the Ségre-Silberberg effect is explained within the micropolar theory [7].

Due to the identity $\mathrm{rot}\;\omega \times b=2{\left(\nabla \omega \right)}_{a}\cdot b$, $\forall b\in R^{3}$, it follows from (68) and (69) that

$$\left(\rho +\frac{D_{\omega }^{2}{\left|\omega_{r}\right|}^{2}}{4\gamma }\right)l=-D\nabla c-D_{p}\nabla p-\frac{D_{\omega }}{2\gamma }N_{a}\cdot \omega_{r}$$

in agreement with the definition of rotary diffusion [37]: “Just as the translational diffusion coefficient is calculated in terms of the drag force, so the rotary diffusion coefficient is expressed in terms of the moment of the forces on a particle executing a rotary movement.”

It follows from (9) that for steady flows the mass conservation law becomes divv=0.

With the above definitions, we arrive at the following conservation laws for steady flows:

(70) div(ρv⊗v)=−∇p+divS+ρf,

(71) Jdiv[ω⊗(ρcv+l)]=divN−ϵ:S,

(72) div(ρcv+l)=0,

with tensors *S*, *N* and the flux l given by Equations (51),(69) and (68) respectively.

For the flows in a concentric-cylinder Couette geometry, we calculate that

$$l=le_{r},\;\rho l=-D\frac{\partial c}{\partial r^{\prime }}-D_{p}\frac{\partial p}{\partial r^{\prime }}+\frac{D_{\omega }\omega_{r}}{2}\frac{\partial \omega }{\partial r^{\prime }}.$$

Under the assumption that c=c(r), we arrive at the formula $\nabla c=e_{r}\partial c/\partial r$. Due to equation divv=0, we obtain that, for the rotation flows, the equation div(ρcv)=0 holds. Now, it follows from (72) that

$$0=\mathrm{div}\;l=\frac{1}{r}\frac{\partial (rl)}{\partial r}\;\mathrm{and}\;rl=\mathrm{const}.$$

At the same time, the now-flow boundary conditions ${l\cdot n|}_{R_{i}}=0$ imply that l=0 and l=0. Thus, the particles mass concentration obeys the equation l=0 or

$$\frac{\partial c}{\partial r}=-\frac{D_{p}}{D}\frac{\partial p}{\partial r}+\frac{D_{\omega }\omega_{r}}{2D}\frac{\partial \omega }{\partial r}.$$

Due to (37), the latter equation can be written as

(73) $$\frac{\partial c}{\partial r}=-\frac{D_{p}}{D}\frac{\rho v^{2}}{r}+\frac{D_{\omega }}{2D}\left(\omega -\frac{1}{2}\left(\frac{\partial v}{\partial r}+\frac{v}{r}\right)\right)\frac{\partial \omega }{\partial r}.$$

In what follows, we use the representations $D_{p}=D_{p}^{*}c\left(1-c\right)$, $D_{\omega }=D_{\omega }^{*}c\left(1-c\right)$ since both $D_{p}$ and $D_{\omega }$ vanish at c=0 and c=1. Given a mean value $c_{0}$ of *c*, we set the following condition:

(74) $$\int_{R_{1}}^{R_{2}}rc\left(r\right)\;dr=\frac{c_{0}\left(R_{2}^{2}-R_{1}^{2}\right)}{2}.$$

As for the particle migration, there is one more approach based on the Fick law. This is known as the Lamm equation for a highly disperse colloidal solution enclosed in a wedge-shape cell rotating at an angular velocity Ω about an axis coinciding with the apex of the wedge [5].

Let us return to Equation (73). One more consequence of the equality l=0 is that the rheological Equation (69) reduces to N=2γA in the Couette geometry.

Let us summarize the mathematical model. We look for functions $S_{r\varphi },S_{\varphi r},\;N_{zr},v,\omega ,c,$ obeying the Equations (35), (36), (38)–(41) and (73), with the given function ε(c).

We introduce dimensionless parameters

$$\rho_{0}=\frac{{\bar{\rho }}_{s}}{{\bar{\rho }}_{f}},\;D_{1}=\frac{D_{p}^{*}R_{1}^{2}\Omega_{0}^{2}{\bar{\rho }}_{s}}{D},\;D_{2}=\frac{D_{\omega }^{*}\Omega_{0}^{2}}{2D}.$$

In new notations,

$$\varepsilon \left(c\right)={\left(1-\frac{c/\phi_{*}}{c+\rho_{0}\left(1-c\right)}\right)}^{-E\phi_{*}}-1.$$

Let us pass to dimensionless variables. Then, Equations (73) and (74) become

(75) $$\frac{1}{c(1-c)}\frac{\partial^{\prime }c}{\partial r^{\prime }}=-\frac{D_{1}v^{\prime 2}}{r^{\prime }\left(c+\rho_{0}\left(1-c\right)\right)}+D_{2}\frac{\partial^{\prime }\omega^{\prime }}{\partial r^{\prime }}\left(\omega^{\prime }-\frac{\partial^{\prime }v^{\prime }/\partial r^{\prime }+v^{\prime }/r^{\prime }}{2}\right),$$

(76) $$\int_{1}^{a}r^{\prime }c\left(r^{\prime }\right)\;dr^{\prime }=\frac{c_{0}\left(a^{2}-1\right)}{2},\;a=\frac{R_{2}}{R_{1}}.$$

We summarize the governing equations as follows. We look for the dimensionless functions $v^{\prime }\left(r^{\prime }\right)$, $\omega^{\prime }\left(r^{\prime }\right)$ and $c(r^{\prime })$ which satisfy Equations (43)–(50), (75) and (76). Observe that these equations are not decoupled since the relative viscosity ε(c) in Equations (44) and (45) depends on the particle concentration *c*.

**Figure 11:**
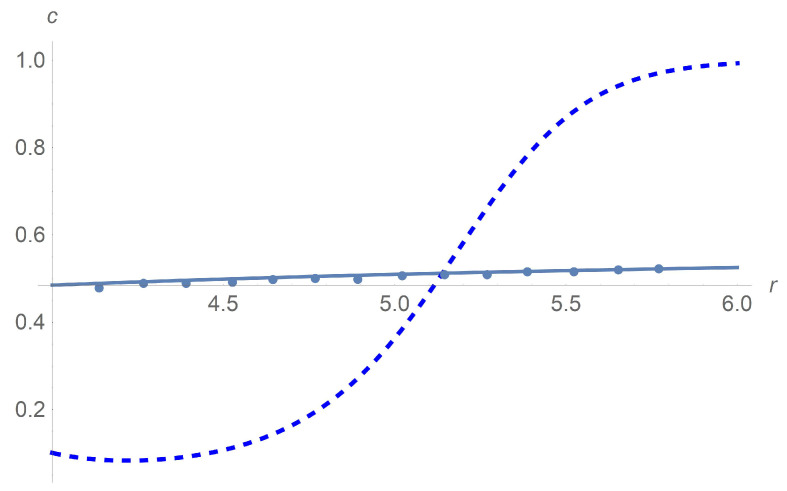
Profiles of mass concentration *c*. Both the solid line that is based on calculations and dots standing for experimental data [9] correspond to $\Omega =10^{2}\;\mathrm{rpm}$. Agreement between calculations and experiment is achieved by the choice $D_{\omega }^{*}/\left(2D\right)=5\times 10^{-5}\;\left[s^{2}\right]$. The dashed line corresponding to calculations reveals the sedimentation effect when we increase Ω to $14\times 10^{3}\;\left[\mathrm{rpm}\right]$.

Figure 11 depicts results of calculations of concentration along the radial variable. We apply the Wolfram Mathematica solver for ordinary differential equations. Agreement between calculations and experiment [9] is achieved for $\Omega =10^{2}\;\mathrm{rpm}$ with the choice $D_{\omega }^{*}/\left(2D\right)=5\times 10^{-5}\;\left[s^{2}\right]$. The sedimentation effect happens when we increase Ω to $14\times 10^{3}\;\left[\mathrm{rpm}\right]$. We prove that such an effect is due to the rotational diffusion $D_{\omega }$ since the particle separation does not happen when $D_{\omega }=0$.

**Remark** **2.** *It is known that, for many practical purposes, the polymers or colloidal particles can be regarded as rigid particles. Examples are triblock Janus particles which can be modeled as cross-linked polystyrene spheres whose poles are patched with sticky alkyl groups, and their middle band is covered with negative charges [38]. This is why the above results on rotational sedimentation can also be applied to polymer flows. As was proved by Svedberg, such flows are of great importance in the studies of the polymer structure. Our contribution is that we propose an alternative approach to the Lamm equation based on the empirical notion of the sedimentation coefficient [6]. The advantages are that we apply the conservation laws of continuum mechanics and take into account the shape of the particles. Moreover, we prove that the polymer sedimentation is due to its rotation. This result suggests a new direction of laboratory studies on polymer flows.*

## 7. Discussion

We address the rotational sedimentation of particles for steady flows of yield stress granular fluids in a concentric-cylinder Couette geometry. Apart from the Lamm equation approach, we do not use the empirical sedimentation coefficient. Instead, we apply conservation laws of the micro-polar equations which allow for particle rotation. We prove that it is due to the rotational diffusion that the particle sedimentation occurs at high angular velocity of the Couette cell inner cylinder. To validate the mathematical model, we perform a comparison with published data of measurements by choosing the relative viscosity related with the particle rotation. First, we justify analytically this choice for dilute suspensions starting from the Einstein correlation for the apparent viscosity. As for dense suspensions, we apply the Krieger–Douhgerty empirical closure for the apparent viscosity. Though we performed calculations for steady flows, the developed approach allows for unsteady flows and non-spherical particles due to the micro-inertia tensor involved into the angular momentum conservation law.

## Data Availability

Not applicable.
